# Prevalence of Child Maltreatment and Its Association with Parenting Style: A Population Study in Hong Kong

**DOI:** 10.3390/ijerph16071130

**Published:** 2019-03-29

**Authors:** Camilla K. M. Lo, Frederick K. Ho, Rosa S. Wong, Keith T. S. Tung, Winnie W. Y. Tso, Matthew S. P. Ho, Chun Bong Chow, Ko Ling Chan, Patrick Ip

**Affiliations:** 1 Department of Applied Social Sciences, The Hong Kong Polytechnic University, Hong Kong; camilla.lo@polyu.edu.hk; 2 Department of Paediatrics and Adolescent Medicine, The University of Hong Kong, Hong Kong; fredkho@connect.hku.hk (F.K.H.); rosawg@connect.hku.hk (R.S.W.); keith-tung@connect.hku.hk (K.T.S.T.); wytso@hku.hk (W.W.Y.T.); mspho@hku.hk (M.S.P.H.); chowcb@netvigator.com (C.B.C.)

**Keywords:** parenting styles, child maltreatment, Chinese

## Abstract

Previous studies point to a link between parenting style and child maltreatment, but evidence from a Chinese context is lacking. We investigated the association between parenting style and child maltreatment in Hong Kong, and examined whether family socio-economic status and child gender moderate this relationship. Using stratified random sampling, 7585 children in Grade 1 to Grade 3 of 51 schools in Hong Kong were recruited and their parents were invited to complete the questionnaire. The past year weighted prevalence for minor physical abuse, severe/very severe physical abuse, psychological abuse, and neglect were 63.9%, 23.4%, 84.1%, and 23.2%, respectively. Authoritarian parenting was associated with all types of child maltreatment (prevalence ratio (PR) range: 1.10–1.53; *p* < 0.001), whereas authoritative parenting was associated with a lower risk of all types of child maltreatment (PR range: 0.89–0.97; *p* < 0.001). Child maltreatment is prevalent in Hong Kong and is strongly associated with parenting style. The association was significantly stronger among girls and those with higher family socioeconomic status. Education to empower parenting skills may alleviate the burden of child maltreatment.

## 1. Introduction

Child maltreatment, including physical abuse, psychological abuse, and neglect, is a global public health issue. A meta-analysis of 244 self-reported studies from different countries estimated the lifetime prevalence of physical abuse, emotional abuse, physical neglect, and emotional neglect were 22.6%, 36.3%, 16.3%, and 18.4%, respectively [1]. While relatively stable or decreasing trends in child maltreatment have been observed in many Western countries, during the study period between 2003 and 2010, increasing trends were found in Hong Kong, one of the most developed cities in China [2,3]. A previous self-reported study of adolescents aged 12 to 17 years found the preceding year prevalence of psychological abuse, physical abuse, severe physical abuse, and neglect were 57.6%, 14.1%, 4.1%, and 27.4%, respectively [4]. It is known that early exposure to child maltreatment is associated with poorer health outcomes in children [5], yet the prevalence of child maltreatment among younger children in Hong Kong is currently unclear. 

Several risk factors related to parent’s characteristics and family relationships have been shown to have significant effects on child maltreatment [6]. In particular, harsh parenting is often viewed as a condition precedent to child maltreatment and is a focus for critical prevention and intervention programs. According to Darling and Steinberg [7], parenting is a construct encompassing two important elements, parenting practices and parenting style. Parenting practices are specific behaviors that parents employ to socialize their children, whereas parenting styles are a collection of attitudes parents express toward their children, and such attitudes create an emotional environment in which children are raised [7]. In regards to parenting style, it is widely accepted that there are three general styles (authoritative, authoritarian, and permissive) based on two dimensions of responsiveness and demandingness [8]. An authoritative parenting style is characterized by responsiveness to the child but being firm in setting rules and limits; an authoritarian parenting style is characterized by high demandingness and high expectations for absolute obedience; and a permissive parenting style is characterized by placing no limits or rules on the child’s behavior. In Western cultures, authoritative parenting is generally considered the optimal parenting style, whereas permissive and authoritarian parenting styles are regarded as dysfunctional approaches [9]. 

It has been shown that authoritarian parenting style is associated with child abuse potential and actual acts of physical abuse [10,11]. However, these findings mainly derived from studies conducted in the West and may not adequately fit the unique cultural contexts in Asian countries. Parenting among Chinese parents is strongly influenced by Confucian ideologies with emphasis on collectivism, filial piety, education, interdependence, strict discipline and obedience, and respect for parents and elders [12]. Hence, authoritarian parenting, which might seem to be controlling and dictatorial in Western cultures, may carry the notions of caring and training in Chinese culture [13,14]. Existing evidence of the associations between authoritarian parenting and child outcomes in Chinese context has been mixed. Some studies found that authoritarian parenting was associated with negative impacts on social and academic performances in Chinese children [15]. Other studies found that authoritarian parenting was associated with positive impacts on socio-emotional development [16] and academic achievement [17], suggesting that the parental monitoring associated with authoritarian parenting may promote child development in Chinese children. However, studies have yet to explore the specific connection between parenting styles and child maltreatment in a Chinese context, and whether such a relationship is moderated by other factors. 

One possible moderating factor is family socioeconomic status (SES), which is determined by parental income, education, and occupation. Parenting is considered to be strongly influenced by family SES through its effects on parental mental health, parental expectations, and access to resources [18]. It was found that parents in low SES families tended to be harsher and more punitive compared to parents in high SES families who tended to use psychological disciplinary techniques, such as reasoning with their children [19,20]. Hence, it would be reasonable to expect a stronger association between authoritarian parenting and child maltreatment in families with lower SES. Furthermore, the literature indicates that child gender also has an influence on parenting practice as well as on social and school performance. Chinese parents tend to have different expectations for boys and girls, as well as having a greater influence on girls [15]. Thus, child gender may be a potential moderating factor for the relationship between parenting styles and child maltreatment in a Chinese context. 

We investigated the association between parenting style and child maltreatment in Hong Kong, one of the major cities in China and with a mixed of Western and traditional Chinese cultural values. Using a representative sample of Hong Kong Chinese children in Grades 1 to 3, this study aimed to (1) examine the prevalence of child maltreatment in Hong Kong, (2) investigate the association between parenting styles and child maltreatment, and (3) examine the potential moderating factors for this association. We hypothesize that authoritarian parenting is associated with increased child maltreatment, whereas authoritative parenting is associated with less child maltreatment; and child gender and family SES moderate the association between parenting style and child maltreatment.

## 2. Materials and Methods

### 2.1. Study Design and Sample

Parents of 7585 children attending Grades 1 to 3 (ages 6 to 10 years) of primary schools in Hong Kong were recruited into the study. Using stratified random sampling, 84 mainstream primary schools from the five main districts in Hong Kong (Hong Kong Island, West Kowloon, East Kowloon, West New Territories, and East New Territories) were invited for this study, in which 51 (60.7%) agreed to participate. The most common refusal reason was being too busy in schooling activities. No significant differences were found between the participated and refused schools. The number of schools recruited in each district was proportional to its child population. All students in Grades 1 to 3 in each selected school were eligible to participate with written consent from the parents. Students whose parents could not comprehend written Chinese were excluded from the study. 

Ethics approval for the study was obtained from the Institutional Review Board of the University of Hong Kong and the Hospital Authority, Hong Kong West Cluster (Reference number: UW 14-632), and all study design and procedures were ensured to follow the safety protocol strictly. One of the parents or legal guardians who were the primary caregivers of the students provided written informed consent and completed the questionnaire. All participants, including children and their proxies, were explained thoroughly for the purpose of the proposed study and their rights to refuse participation and to withdraw from the study at any time. Anonymity and confidentiality were assured.

### 2.2. Measures

#### 2.2.1. Outcome Measure

The Parent-Child Conflict Tactics Scale (CTS-PC) was used to measure child maltreatment [21]. The CTS-PC contains items that measure physical maltreatment (13 items with subscales ranging from corporal punishment to severe physical assault), psychological aggression (5 items), neglect (5 items), sexual abuse (4 items), and discipline (4 items). The tool was previously translated and validated using Hong Kong data with satisfactory reliability and validity [22]. The tool has been used in various population-based surveys in Hong Kong, which have demonstrated its ability to identify child maltreatment [23].

#### 2.2.2. Predictors

The Parenting Styles and Dimensions Questionnaire (PSDQ) was used to assess the parents’ general parenting style [24]. The original scale has three dimensions (permissive, authoritative, and authoritarian), but the permissive dimension was found to be an unreliable construct for Chinese parents and inappropriate in a Chinese cultural context [25]. Therefore, only the authoritative and authoritarian subscales were used in this study. The authoritative subscale consists of 27 items using a five-point Likert scale, which measure several factors including warmth and involvement, reasoning/induction, democratic participation, and good-natured/easy going. The authoritarian subscale consists of 20 items using a five-point Likert scale, which measure several factors including verbal hostility, corporal punishment, non-reasoning/punitive strategies, and defectiveness. The PSDQ is a psychometrically sound scale for assessing parenting practices [26] and has been validated and used in previous Chinese community studies [27]. 

Principal component analysis was used to identify dimensions of parenting styles. Consistent with previous study [28], two dimensions of parenting style, one corresponding to authoritative and the second corresponding to authoritarian, were identified. Hence, parents in the study were classified into authoritarian and authoritative parenting groups. To further capture the interaction of the two parenting style dimensions, four groups were formed: (1) mainly authoritarian (high authoritarian and low authoritative), (2) uninvolved (low authoritarian and low authoritative), (3) inconsistent (high authoritarian and high authoritative), and (4) mainly authoritative (low authoritarian and high authoritative). 

#### 2.2.3. Moderators

Child gender and family SES and were examined as potential moderating factors. The family SES was assessed using a validated family questionnaire that has been found to be relevant in the Hong Kong context and has demonstrated reasonable correlations with childhood developmental outcomes [29]. The questionnaire items cover parents’ occupation, education level, family income, family structure, and residential district. An aggregated SES index was calculated for each family using principal component analysis, which has been shown to be a valid method in the Hong Kong population [30]. 

### 2.3. Statistical Analysis

Descriptive statistics were used to analyze the demographic characteristics of the study participants, prevalence of the different types of child maltreatment (including physical abuse, psychological abuse, and neglect), and patterns of parenting styles. Weighted prevalence of child maltreatment was calculated according to the age- and sex-specific population based on the most recent by-census in Hong Kong [31]. Poisson regression was used to estimate the prevalence ratio of maltreatment between different parenting styles, with robust ‘sandwich’ estimation of standard error [32] and adjustment for child gender, age, and family SES. The prevalence ratio can be interpreted in a similar way as the risk ratio. Prevalence ratio, instead of risk ratio, was used because risk (incidence) cannot be estimated in cross-sectional studies. Moderation analyses were conducted to examine the potential interaction effects of child gender and family SES with parenting styles. All data analyses were performed using the R statistical package [33]. All two-tailed *p*-values < 0.05 were considered statistically significant.

## 3. Results

Of the 10,274 invitations sent out, 7585 (73.8%) parent–child dyads agreed to participate in the study and returned completed questionnaires. The characteristics of the participants are presented in Table 1. The mean age of the 7585 children was 8.15 years (SD = 0.88) and 54.3% were male. The mean family income was USD 4077.37 (SD = 3670.62).

The parenting style patterns among the study participants are shown in Table 1. In terms of parenting styles, 23.81% of the parents were characterized as mainly authoritarian (high authoritarian and low authoritative), 20.65% were uninvolved (low authoritarian and low authoritative), 20.4% were inconsistent (high authoritarian and high authoritative), and 35.15% were mainly authoritative (low authoritarian and high authoritative).

The estimated past year prevalence of child maltreatment in Hong Kong is shown in Table 2. Prevalence of minor physical abuse, severe abuse, psychological abuse, and neglect were 63.9%, 23.4%, 84.1%, and 23.2%, respectively. The prevalence of physical abuse and psychological abuse was higher among boys than girls. The prevalence of child neglect was higher among girls than boys. The prevalence of physical abuse and psychological abuse decreased with age, whereas the prevalence of child neglect increased with age.

The associations between the role of parenting style and child maltreatment are shown in Table 3. After controlling for age, gender, and family SES, authoritarian parenting style was a consistent risk factor for various types of child maltreatment including severe physical abuse (prevalence ratio (PR): 1.53; 95% CI: 1.45, 1.62; *p* < 0.001), minor physical abuse (PR: 1.23; 95% CI: 1.20, 1.26; *p* < 0.001), psychological abuse (PR: 1.10; 95% CI: 1.09, 1.12; *p* < 0.001), and neglect (PR: 1.26; 95% CI: 1.18, 1.34; *p* < 0.001). Authoritative parenting style was a protective factor for different types of child maltreatment including severe physical abuse (PR: 0.89; 95% CI: 0.84, 0.95; *p* < 0.001), minor physical abuse (PR: 0.94; 95% CI: 0.91, 0.97; *p* < 0.001), psychological abuse (PR: 0.97; 95% CI: 0.96, 0.99; *p* < 0.001), and neglect (PR: 0.90; 95% CI: 0.84, 0.96; *p* = 0.003) after adjusting for confounding variables.

**Figure 1:**
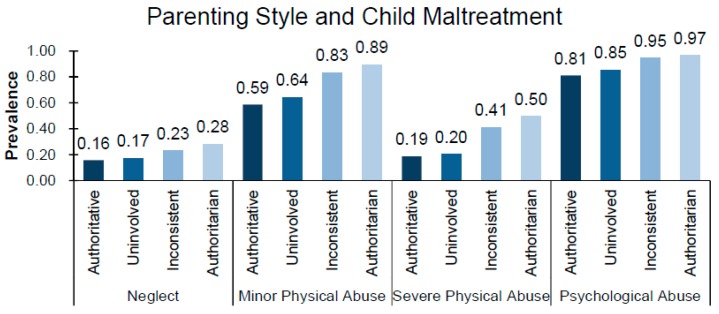
Association between categories of parenting style and child maltreatment.

The prevalence ratios of different types of child maltreatment according to the parenting style are shown in Figure 1. Parents with mainly authoritarian parenting style were more likely to perpetrate different types of child maltreatment (PR range: 0.28–0.97), followed by inconsistent parenting (PR range: 0.23–0.95), uninvolved parenting (PR range: 0.17–0.85), and mainly authoritative parenting (PR range: 0.16–0.81). 

**Figure 2:**
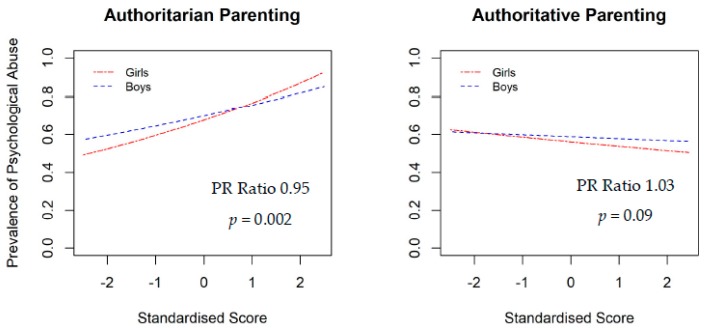
Moderation effect of child gender on the association between parenting style and child maltreatment.

**Figure 3:**
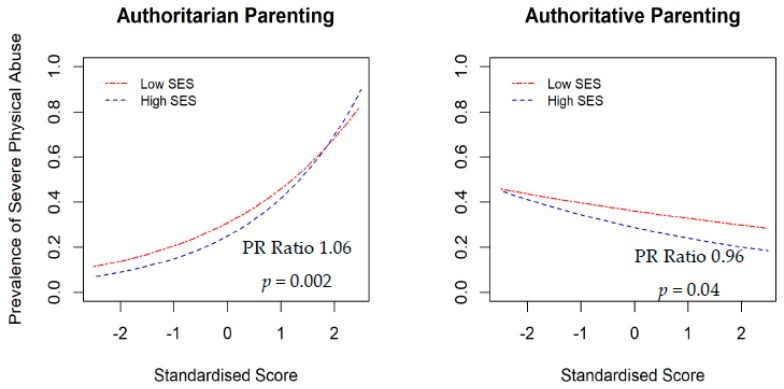
Moderation effect of family socioeconomic status on the association between parenting style and child maltreatment.

The moderating effects of family SES and child gender were also examined in the study. The interaction effects between parenting styles and child gender for psychological abuse (Figure 2) indicated both authoritarian and authoritative parenting styles had stronger effects on psychological abuse in girls than boys. The moderating effects between authoritarian and authoritative parenting styles and SES for severe physical abuse (Figure 3) indicated that parenting styles had stronger effects in high SES families. Specifically, child physical abuse was more prevalent among high SES families with authoritarian parenting than those in low SES families.

## 4. Discussion

### 4.1. Study Findings

This study adds to the literature on the association between parenting styles and child maltreatment in a Chinese context and provides a more accurate estimation of the prevalence of different types of child maltreatment in young children in Hong Kong. Using a large and representative sample of Chinese children and their parents, this study showed that child maltreatment is highly prevalent among young children in Hong Kong as measured by CTS-PC proxy-report of child maltreatment. The high past year weighted prevalence of psychological abuse (84.1%) is particularly worrying as it is strongly associated lower self-esteem, higher levels of depression, and a greater sense of helplessness [34], and is predictive of physical abuse [35]. 

Contrary to the previous understanding that authoritarian parenting style is commonly endorsed and adopted by Chinese parents, this study showed that authoritative parenting was more common than authoritarian parenting, with 35.15% of parents classified as mainly authoritative (low authoritarian and high authoritative) compared with 23.81% as mainly authoritarian (high authoritarian and low authoritative). Consistent with findings in the West, this study showed strong associations between parenting styles and child maltreatment. Parents with authoritarian parenting styles were more likely to perpetrate different types of child maltreatment, whereas authoritative parenting was a protective factor for child maltreatment. Although authoritarian parenting may be beneficial to children in some aspects [15], the findings from this study suggest the potential positive outcomes may come at a cost to the child’s welfare. 

This study found that the associations between parenting style and child maltreatment may depend upon the family SES and child’s gender. Although child physical abuse was prevalent in both high SES and low SES families, child physical abuse was more prevalent among high SES families with authoritarian parenting than those in low SES families. One possible explanation for this observation is that parents in high SES families tend to have higher expectations of their children, particularly in educational attainment [36], and would exert greater pressure on their children compared to parents in lower SES families [20]. Such authoritarian parenting styles together with higher expectations may have a greater influence on child physical abuse in high SES families. The interaction effect was not found for other types of child maltreatment, including neglect and physical abuse. One possible reason for this observation is that these two types of maltreatment are more likely to be associated with parents’ emotional and psychological problems [2,37]. In addition, this study found that child gender moderated the association between parenting style and child maltreatment, particularly for psychological abuse. This observation may be explained by the difference in parental expectations for boys and girls. In particular, parents’ expectations for girls are influenced by the chastity virtue in Chinese culture. Parents tend to exert higher behavioral control and demandingness, and foster dependency in girls than in boys [38]. The relationship between parents and daughters therefore tend to be more closely tied. In addition, girls are less likely to experience physical abuse compared to boys [2]. These reasons together may explain why child gender moderated the association between parenting style and psychological abuse. However, these ideas are speculative in nature and would have to be tested in further research. 

### 4.2. Implications

This study showed that dysfunctional parenting styles elevate the risk of child maltreatment. Strong prevention measures to reduce harmful parenting are warranted. A previous meta-analysis showed that parenting programs were effective in reducing child maltreatment by changing parental risk factors such as parental stress and mental health, parenting attitudes and skills, knowledge about child development, and parental sensitivity [39]. It is likely that parents will be more willing to discuss their parenting styles and practices rather than their maltreatment acts toward their children. Engaging parents to discuss their attitudes toward raising their children will provide an opportunity for early identification and timely intervention for problematic parenting that could potentially increase the risk for child maltreatment. Although there are many available parenting programs that are beneficial to parents and their children, many parents do not participate in such interventions [40]. Increased effort needs to be made to scale up these parenting programs as a preventive strategy for child maltreatment. For example, delivery of parent training via mobile phones or tablets may offer an alternative to traditional face-to-face delivery to enhance service accessibly for parents. A previous study has demonstrated that a tablet-based parenting program yielded comparable effect sizes with a face-to-face parenting program [41]. Chinese Parents who endorse an authoritarian parenting style may demand strict obedience from children out of the notion of training and discipline rather than harm. In terms of practice, professionals working with Chinese parents should engage and respectfully discuss with them about the potential harms of authoritarian parenting. An individual’s parenting style is strongly influenced by how the individual was raised by their own caregivers [42]. Interventions that facilitate parents in reflecting on how caregivers influence parenting would be helpful in altering parenting styles. 

### 4.3. Limitations

This study has several limitations that need to be acknowledged. The measures used in this study were proxy-reported and may be subject to recall bias and social desirability, particularly parents may under-report child maltreatment and negative aspects of parenting [43,44]. Hence, the associations between parenting styles and child maltreatment may have been underestimated. Further studies using multiple informants will help address this limitation. As this study was cross-sectional in nature, the causal relationship between the variables cannot be established. 

## 5. Conclusions

This study examined the association between parenting styles and child maltreatment in a representative sample of Chinese children in Hong Kong. The study showed that child maltreatment is prevalent among young children in Hong Kong. We found authoritarian parenting style was positively associated with different types of child maltreatment, whereas authoritative parenting style was a protective factor for child maltreatment. Family SES and child gender moderated the relationship between parenting style and child maltreatment. Contrary to previous findings of the benefits of authoritarian parenting on Chinese children, this study revealed that such parenting is actually harmful to Chinese children.

## Figures and Tables

**Table 1:** Participant characteristics (*n* = 7585).

Characteristics	Mean (SD)/N (%)
Boys	4119 (54.3)
Age, mean (SD), years	8.15 (0.88)
Family income, mean (SD), HKD	31,803.45 (28,630.86)
Past year child maltreatment	
Minor physical abuse	4268 (56.3)
Severe/very severe physical abuse	1602 (21.1)
Psychological abuse	5646 (74.4)
Neglect	1623 (21.4)
Parenting style scores	
Authoritative style, mean (SD), range = 1–5	3.72 (0.55)
Authoritarian style, mean (SD), range = 1–5	1.83 (0.45)
Parenting style	
Mainly authoritarian	23.81%
Mainly authoritative	35.15%
Uninvolved	20.65%
Inconsistent	20.40%

**Table 2:** Weighted prevalence of child maltreatment.

	Weighted Prevalence (%)			
	Physical Abuse		Psychological Abuse	Child Neglect
	Minor	Severe or Very Severe		
**Overall**	63.9	23.4	84.1	23.2
**Gender**				
Girls	59.1	19.8	81.8	24.1
Boys	68.4	26.8	86.2	22.3
**Age in years**				
6	69.5	26.1	86.3	21.0
7	65.8	24.1	85.5	20.8
8	62.8	23.4	83.6	24.5
9	56.8	19.6	80.5	26.7

**Table 3:** Association between parenting style and child maltreatment.

	Age and Gender Adjusted		SES Additionally Adjusted	
	PR (95% CI)	*p*	PR (95% CI)	*p*
**Neglect**				
Authoritarian	1.31 (1.23, 1.39)	<0.001 ***	1.26 (1.18, 1.34)	<0.001 ***
Authoritative	0.85 (0.80, 0.90)	<0.001 ***	0.90 (0.84, 0.96)	0.003 **
**Minor physical abuse**				
Authoritarian	1.24 (1.21, 1.27)	<0.001 ***	1.23 (1.20, 1.26)	<0.001 ***
Authoritative	0.92 (0.90, 0.95)	<0.001 ***	0.94 (0.91, 0.97)	<0.001 ***
**Severe/very severe physical abuse**				
Authoritarian	1.58 (1.50, 1.66)	<0.001 ***	1.53 (1.45, 1.62)	<0.001 ***
Authoritative	0.85 (0.80, 0.90)	<0.001 ***	0.89 (0.84, 0.95)	<0.001 ***
**Psychological abuse**				
Authoritarian	1.11 (1.09, 1.12)	<0.001 ***	1.10 (1.09, 1.12)	<0.001 ***
Authoritative	0.97 (0.95, 0.98)	<0.001 ***	0.97 (0.96, 0.99)	<0.001 ***

Parenting style scores were standardized to their mean and SD. PR: prevalence ratio, a measure of association in cross-sectional studies. *** *p* < 0.001, ** *p* < 0.05.
